# The P2X7 Receptor Primes IL-1β and the NLRP3 Inflammasome in Astrocytes Exposed to Mechanical Strain

**DOI:** 10.3389/fncel.2017.00227

**Published:** 2017-08-08

**Authors:** Farraj Albalawi, Wennan Lu, Jonathan M. Beckel, Jason C. Lim, Stuart A. McCaughey, Claire H. Mitchell

**Affiliations:** ^1^Department of Anatomy and Cell Biology, University of Pennsylvania, Philadelphia PA, United States; ^2^Department of Orthodontics, University of Pennsylvania, Philadelphia PA, United States; ^3^Department of Pharmacology and Chemical Biology, Pittsburgh University, Pittsburgh PA, United States; ^4^Department of Ophthalmology, University of Pennsylvania, Philadelphia PA, United States; ^5^Department of Physiology, University of Pennsylvania, Philadelphia PA, United States

**Keywords:** IL-1β, astrocytes, glaucoma, pannexin, ATP release, NFκB, caspase 1, NLRP3

## Abstract

Inflammatory responses play a key role in many neural pathologies, with localized signaling from the non-immune cells making critical contributions. The NLRP3 inflammasome is an important component of innate immune signaling and can link neural insult to chronic inflammation. The NLRP3 inflammasome requires two stages to contribute: priming and activation. The priming stage involves upregulation of inflammasome components while the activation stage results in the assembly and activation of the inflammasome complex. The priming step can be rate limiting and can connect insult to chronic inflammation, but our knowledge of the signals that regulate NLRP3 inflammasome priming in sterile inflammation is limited. This study examined the link between mechanical strain and inflammasome priming in neural systems. Transient non-ischemic elevation of intraocular pressure increased mRNA for inflammasome components *IL-1*β*, NLRP3, ASC*, and *CASP1* in rat and mouse retinas. The elevation was greater 1 day after the insult, with the rise in IL-1β most pronounced. The P2X7 receptor was implicated in the mechanosensitive priming of IL-1β mRNA *in vivo*, as the antagonist Brilliant Blue G (BBG) blocked the increased expression, the agonist BzATP mimicked the pressure-dependent rise in IL-1β, and the rise was absent in P2X7 knockout mice. *In vitro* measurements from optic nerve head astrocytes demonstrated an increased expression of *IL-1*β following stretch or swelling. This increase in *IL-1*β was eliminated by degradation of extracellular ATP with apyrase, or by the block of pannexin hemichannels with carbenoxolone, probenecid, or 10panx1 peptide. The rise in *IL-1*β expression was also blocked by P2X7 receptor antagonists BBG, A839977 or A740003. The rise in *IL-1*β was prevented by blocking transcription factor NFκB with Bay 11-7082, while the swelling-dependent fall in NFκB inhibitor IκB-α was reduced by A839977 and in P2X7 knockout mice. In summary, mechanical trauma to the retina primed NLRP3 inflammasome components, but only if there was ATP release through pannexin hemichannels, and autostimulation of the P2X7 receptor. As the P2X7 receptor can also trigger stage two of inflammasome assembly and activation, the P2X7 receptor may have a central role in linking mechanical strain to neuroinflammation.

## Introduction

Mechanical trauma can induce complex pathological changes to neural tissue via inflammation (Corps et al., 2015; Heppner et al., 2015). While recruitment of immune cells to the injured region can contribute, localized inflammatory signaling between glia and neurons can also initiate or enhance inflammatory damage. The NLRP3 inflammasome is a key component of the localized innate immune system, leading to the cleavage and release of pro-inflammatory cytokines (Rathinam et al., 2012), and it has been implicated in neural disorders associated with mechanical strain or elevated pressure (Walsh et al., 2014), including traumatic brain injury, encephalitis, and glaucoma (Kaushik et al., 2012; Liu et al., 2013; Chi et al., 2014, 2015).

The involvement of the NLRP3 inflammasome is a two-step process. In the first stage, referred to as the priming step, expression of inflammasome components such as pro-IL-1β and NLRP3 is increased at the transcriptional and translational level (Mariathasan et al., 2006; Patel et al., 2017). This priming stage can be the rate-limiting step in inflammatory responses and may connect the initial insult to chronic inflammation. During the second stage, inflammasome components are assembled and activated, turning on caspase 1 which subsequently catalyzes the maturation of cytokines IL-1β and IL-18 (Stutz et al., 2009). This later step has been linked to efflux of K^+^ through the P2X7 purinergic receptor (Mariathasan et al., 2006; Petrilli et al., 2007; Bernier, 2012; Karmakar et al., 2016), even for activation associated with lysosomal rupture (Muñoz-Planillo et al., 2013), and can be mimicked by the K^+^ ionophore nigericin (Perregaux and Gabel, 1994). While activation of the NLRP3 inflammasome has been the subject of intense investigation (e.g., Guo et al., 2015; Yilmaz and Lee, 2015; Freeman and Ting, 2016), the signals leading to inflammasome priming are less well understood. Standard models attribute priming to microbial molecules or other toll-like receptor agonists that are rarely detected in sterile neural environments.

The central role of aberrant purinergic signaling in the neuropathology triggered by mechanical strain has been outlined for the retina (Mitchell et al., 2009). In astrocytes isolated from the optic nerve head, moderate strain leads to a release of ATP through pannexin hemichannels (Beckel et al., 2014). This released ATP then autostimulates P2X7 receptors on these astrocytes to regulate cytoplasmic Ca^2+^ and other physiological responses. Expression of pannexins is increased *in vitro* by cell stretch and *in vivo* in a model of chronic intraocular pressure (IOP) elevation, consistent with a role for ATP release in the neural response to sustained mechanical strain. Elevated extracellular ATP was confirmed in primate, rat, and mouse models of chronic IOP elevation (Lu et al., 2015) and detected in the eyes of humans with chronic glaucoma (Li et al., 2011).

This study asks whether extracellular ATP release through pannexins and autostimulation of the P2X7 receptor are involved in the priming of the NLRP3 inflammasome. The data are consistent with a role for the P2X7 receptor in priming IL-1β and NLRP3 in retina following activation of NFκB in optic nerve head astrocytes. This identifies a new pathway for priming the inflammasome in sterile neural environments subject to mechanical strain.

## Materials and Methods

### Animal Care and Use

All procedures were performed in strict accordance with the National Research Council’s “Guide for the Care and Use of Laboratory Animals” and were approved by the University of Pennsylvania Institutional Animal Care and Use Committee (IACUC). All animals were housed in temperature-controlled rooms on a 12:12 light:dark cycle with food and water *ad libitum*. Long–Evans and Sprague Dawley rats (Harlan Laboratories/Envigo, Frederick, MD, United States), and mice (C57BL/6J wild type and P2X7^-/-^ knock out) of both sexes were utilized. Both the wild type C57BL/6J and the P2X7^-/-^ B6.129P2-P2rx7^tm1Gab^/J Pfizer mice were from Jackson Laboratories (Bar Harbor, ME, United States). Tg-Myoc^Y 437H^ mice exhibiting a moderate rise in IOP were received as a gift from Zode et al. (2011). Previous characterization of the mice in our laboratory confirmed the moderate, sustained elevation of IOP and loss of peripheral ganglion cells in Tg-Myoc^Y 437H^ mice (Lu et al., 2015).

### Controlled Elevation of IOP Model

The IOP was elevated in adult Sprague-Dawley rats as previously reported (Lu et al., 2017) based on the Control Elevation of IOP (CEI) protocol developed by Morrison et al. (2010, 2014). This procedure separates the effects of increased ocular pressure from cell death to enable a focus specifically on the consequences of mechanical strain. Pressures were selected so that retinal blood flow was maintained and ischemia avoided; studies suggest this protocol leads to minimal loss of neurons (Crowston et al., 2015; Lu et al., 2017).

After receiving 2 mg/kg meloxicam, rats were deeply anesthetized with 1.5% isoflurane or intraperitoneal injection of ketamine (80 mg/kg) and xylazine (10 mg/kg). After administration of proparacaine (0.5%) and tropicamide (0.5–1%), one eye was cannulated with a 27-gauge shielded wing needle (Becton Dickinson, NJ) inserted into the anterior chamber, connected to a 20 ml syringe filled with sterile phosphate buffered saline (PBS). IOP was increased to 50–60 mmHg by elevating the reservoir to the appropriate height; blood flow through the retina was maintained throughout to avoid ischemic complications. Slightly higher pressures were needed with isoflurane. The contralateral eye without cannulation served as a normotensive control. After 4 h, IOP was returned to normal, the needle removed and 0.3% gentamicin ointment or 0.5% erythromycin applied to the cornea. In rat experiments, tissue was obtained as soon as the IOP was returned to baseline. Experiments on mice were performed using procedures similar to those described elsewhere (Crowston et al., 2015). Mice were anesthetized with 1.5% isoflurane, and IOP was increased to 60 mmHg for 4 h, pressure was returned to normal, the needle removed and 0.5% erythromycin applied to the cornea. Mice were either sacrificed immediately or 22 h later to examine the time dependence of gene upregulation.

### Intravitreous Injection

Intravitreal injections were performed in rat eyes under a dissecting microscope using a micropipette connected to a microsyringe (Drummond Scientific Co., Broomall, PA, United States) as described (Hu et al., 2010). A glass pipette filled with P2X7 receptor antagonist Brilliant Blue G (BBG, 0.8%) dissolved in saline was passed through the superior nasal region of sclera into the vitreous cavity, ∼1 mm from the limbus, with a total volume of 5 μl injected over a 30 s time period. The antagonist was delivered 1–3 days before IOP elevation. C57BL/6J wild type mice were injected with either P2X7 receptor agonist BzATP (2 μl, 250 μM) or sterile saline.

### Astrocyte Cell Culture

Primary rat optic nerve head astrocyte cultures were grown based on a protocol modified from Mandal et al. (2009). The optic nerve proximal to the sclera, defined as the optic nerve head, was obtained from rat pups PD3-5 of both genders; rat astrocytes grew much more efficiently when obtained from neonatal material. This optic nerve head tissue was digested for 1 h using 0.25% trypsin (Invitrogen), with periodic trituration to create a cell suspension. Cells were washed once with Dulbecco’s modified Eagle’s medium (DMEM)/F12 containing 10% of fetal bovine serum (FBS), re-suspended in DMEM/F12, 10% FBS, 1% penicillin/streptomycin, and 25 ng/mL epidermal growth factor (#E4127, Sigma–Aldrich) plated on 35 mm culture dishes and grown at 37°C, 5.5% CO_2_. Cultures were found to contain >99% astrocytes, as defined by glial fibrillary acidic protein (GFAP) immunofluorescence staining. Cells were generally used at passages 2–5. Mouse optic nerve head astrocyte tissue was collected from 3-month-old C57BL/6J and P2X7^-/-^ mice of both genders using a similar protocol but with only 35 min of trypsin incubation; mouse neonates were not used as the tissues were too small to be handled appropriately.

### Swelling Isolated Astrocytes

Rat and mouse optic nerve head astrocytes were subcultured onto plastic 6-well plates and grown until confluent. Cells were incubated in control isotonic solution containing (in mM) 105 NaCl, 5 KCl, 4 NaHEPES, 6 HEPES acid, 1.3 CaCl_2_, 5 glucose, 5 NaHCO_3_ and 60 mannitol, pH 7.4 or in solution made 30% hypotonic solution by addition of dH_2_0, for 4 h in the tissue culture incubator. Cells were pretreated with inhibitors Bay 11-7082 (4 μM), Brilliant blue G (BBG, 10 μM), A839977 (50 nM, Tocris Bioscience), A740003 (5 μM, Tocris Bioscience), carbenoxolone (10 μM, #C4790), probenecid (1 mM, #P8761) or ^10^Panx1 and scrambled peptide (100 μM, #3348 and #3708, respectively, Tocris Bioscience) for 1 h before adding test solutions. RNA was extracted immediately after the 4 h treatment.

### *In Vitro* Stretch Experiments

Isolated rat optic nerve head astrocytes were plated on silicone substrates coated with collagen 1 (Flexcell biaxial six-well plate #BF-3001C, Flexcell International Corp.) for 6–7 days until confluent. After replacing medium with isotonic solution, cells were exposed to square strain of 16% at 0.3 Hz for 4 h in the tissue culture incubator using a vacuum with the Flexcell FX-5000 Tension System (Flexcell International Corp.). Control cells were cultured on similar plates and kept in the same incubator without stretch. RNA was extracted immediately after the 4 h stretch period.

### Quantitative PCR

RNA was extracted from astrocytes or retinas by homogenizing in 1 ml TRIzol reagent (Invitrogen), with total RNA purified using a RNeasy mini kit (#79254, Qiagen, Inc., Germantown, MD, United States). RNA concentration and purity were assessed using a Nanodrop spectrophotometer (Thermo Scientific). cDNA was synthesized from 1 μg of total RNA per reaction using the High Capacity cDNA Reverse Transcription Kit (#4368814, Applied Biosystems) at 25°C for 10 min, 37°C for 120 min and terminated at 85°C for 5 min. The Quantitative Polymerase Chain Reaction (qPCR) was performed using SYBR Green and the 7300 RealTimePCR system (Applied Biosystems Corp.), starting with 50°C for 2 min and 95°C for 10 min, followed by 40 cycles at 95°C for 15 s and 60°C for 1 min, and concluding with 15 s at 95°C, 60°C for 1 min and 95°C for 15 s to ensure a single product on melting curves; 0.5 μl of cDNA was used per well, except for *in vitro* analysis of *IL-1*β in which 1 μl was used. All data were analyzed using the ΔΔ*C*t approach as described previously (Coffey et al., 2014). Primers used are described in **Table 1**.

**Table 1:** Primers used for qPCR.

Gene name	GenBank accession	Forward primer (5′–3′)	Reverse primer (5′–3′)	Size (bp)
Rat IL-1β 1	NM_031512.2	GGGATTTTGTCGTTGCTTGT	CTGTGACTCGTGGGATGATG	211
Rat IL-1β 2	NM_031512.2	CACCTCTCAAGCAGAGCACAG	GGGTTCCATGGTGAAGTCAAC	83
Rat NLRP3	NM_001191642	CCATGAGCTCCCTTAAGCTG	TTGCACAGGATCTTGCAGAC	283
Rat CASP1	NM_012762	TATGGAAAAGGCACGAGACC	CAGCTGATGGACCTGACTGA	137
Rat ASC	NM_172322.1	CCCATAGACCTCACTGATAAAC	AGAGCATCCAGCAA ACCA	260
Rat IL-18	NM_019165.1	GGACTGGCTGTGACCCTATC	TGTCCTGGCACACGTTTCTG	152
Mouse IL-1β 1	NM_008361.4	GAAGATGGAAAAACGGTTTG	GTACCAGTTGGGGAACTCTGC	85
Mouse IL-1β 2	NM_008361.4	CAAGCTTCCTTGTGCAAGTGTCTG	AGGACAGCCCAGGTCAAAGGTT	161
Mouse NLRP3	NM_145827.3	AGAGCCTACAGTTGGGTGAAATG	CCACGCCTACCAGGAAATCTC	116
Mouse CASP1	NM_009807.2	TGGTCTTGTGACTTGGAGGA	TGGCTTCTTATTGGCACGAT	172
Mouse ASC	NM_023258.4	GGAGTCGTATGGCTTGGAGC	CGTCCACTTCTGTGACCCTG	204
Mouse IL-18	NM_008360.1	CAGTGAACCCCAGACCAGAC	TGTTGTGTCCTGGAACACGT	212
Mouse P2X7R	NM_001284402.1	TGGAACCCAAGCCGACGTTGA	CTCGGGCTGTCCCCGGACTT	250
Mouse Bax	NM_007527.3	TGCAGAGGATGATTGCTGAC	GATCAGCTCGGGCACTTTAG	154
GAPDH	NM_017008	TCACCACCATGGAGAAGGC	GCTAAGCAGTTGGTGGTGCA	195

For the PCR gel used in genotyping, RNA was extracted from confluent wild type and P2X7^-/-^ mouse optic nerve head astrocytes and converted to cDNA as above. PCR amplification reaction included 10 μl REDExtract-N-Amp PCR Reaction mixture (#XNATS, Sigma–Aldrich) with 4 μl of the cDNA, 2 μl H2O and 2 μl of the P2X7 receptor primer (10 μM) (**Table 1**). cDNA was denatured at 94°C for 3 min, followed by 35 PCR cycles. Each consists of three steps: 94°C for 45 s, 65°C for 1 min, and 72°C for 1 min. Final extension was set at 72°C for 10 min. PCR products were detected by 1% (w/v) agarose gels using a 100 base pair DNA Ladder (#15628-019, Invitrogen).

### Immunocytochemistry

Astrocytes were grown to 80% of confluence, fixed with 4% formaldehyde for 20 min at 25°C, permeabilized with 0.1% Triton X-100 (Bio-Rad, United States) for 15 min then blocked with 20% Superblock (Thermo Fisher Scientific Inc.) in PBS with Tween 20 (Bio-Rad, United States) (PBS/T) for 1 h. Coverslips were incubated with anti-GFAP monoclonal antibody (#MABH360, 1:250: Chemicon Int.) overnight at 4°C, followed by donkey anti-mouse IgG Alexa-Fluor 488 for 60 min (#A21202, 1:500; Invitrogen). For pannexin 1 (panx1), cells were incubated with goat polyclonal (#ab124131, 1:200, Abcam), followed by rabbit anti-goat 594 nm; over 80% of astrocytes stained positive for panx1. For P2X7 receptor staining, cells were incubated overnight with rabbit polyclonal (#APR-008, 1:100, Alomone), followed by donkey anti-rabbit IgG Alexa Fluor 488 for 1 h (#A21206, 1:500, Invitrogen); over 80% of astrocytes stained positive for P2X7. Cells were incubated with Alexa Fluor 568 Phalloidin (#A12380, 1:100; Invitrogen) for 15 min. After incubation with DAPI (#D9542, Sigma–Aldrich) for 10 min, coverslips were washed and mounted using SlowFade Gold anti-fade media (Invitrogen). Images were acquired using a Nikon Eclipse microscope (Nikon, United States) and Image-Pro software (Media Cybernetics). ImageJ (Schindelin et al., 2015) was used to subtract background, modify intensity and combine pseudocolored images, with parallel processing for all images. Mouse astrocytes were stained with the same anti-GFAP monoclonal antibody used for rat cells.

For retinal sections, mice were transcardially perfused with 4% formaldehyde in PBS. After enucleation, eyes were fixed with 4% formaldehyde overnight then incubated in 30% sucrose for 2 h. Eyes were embedded in OCT compound (Tissue-Tek #62550-1), cryosectioned at 9 μm and mounted on Colorfrost plus slides (Thermo Fisher #9991001). Sections were fixed with 4% formaldehyde for 10 min, permeabilized with 0.1% Triton-X-100 for 10 min, then blocked with 20% Superblock with PBS/T plus 10% donkey serum. Sections were incubated in 5 μg/ml IL-1β primary antibody (goat polyclonal antibody #AF-401-NA R&D systems, Lot #NP2715111) and anti-GFAP monoclonal antibody (#MABH360, 1:250: Chemicon Int.) overnight at 4°C, followed by secondary donkey anti-goat Alexa555-conjugated antibody (#A21432) and donkey anti-mouse IgG Alexa Fluor 488 for 60 min (#A21202, 1:500; Invitrogen). Images were obtained as described above.

### Immunoblots

Immunoblots were processed as described (Guha et al., 2012; Coffey et al., 2014). In brief, whole retinas or astrocytes grown on six well-plates were washed twice with cold PBS and lysed in RIPA buffer containing 50 mM Tris-HCl, 150 mM NaCl, protease inhibitor cocktail (Complete; Roche Diagnostics, Germany), 1% Triton X-100, 0.1% SDS, and 10% glycerol. Sonicated samples were centrifuged (14,000 *g*, 10 min, 4°C) and quantified using a BCA Protein Assay (Pierce/Thermo Fisher). Protein was separated using conventional SDS-PAGE, and processed standard immunoblot protocols (Lu et al., 2015). Polyvinylidene difluoride blots were incubated with 0.25 μg/ml IL-1β goat polyclonal antibody (#AF-401-NA R&D systems, Lot #NP2715111), IκBα rabbit polyclonal antibody (#9242, 1:1000; Cell Signaling) overnight at 4°C, followed by donkey anti-goat IgG-HRP (1:5000; #sc-2020, Santa Cruz Biotechnology) or rabbit IgG, HRP-linked whole antibody from donkey (1:5000, #NA934, Amersham Bioscience Corp.). Blots were developed using the chemiluminescence detection (ECL detection system; Amersham Biosciences Corp.) and visualized with the ImageQuant LAS 4100 imager and Image Quant software (GE Healthcare Life Sciences).

### ATP Measurement

Rat astrocytes cells were grown to confluence in a 96-well assay plate (#3610, Corning Inc.). Growth medium was replaced with isotonic solution and cells were allowed to recover for 30 min at 37°C before measurements were taken. A bioluminescent luciferin/luciferase assay was used to measure ATP levels (Reigada et al., 2005). The luciferin/luciferase mix (#FLAAM, Sigma–Aldrich) was stored frozen as a stock solution with 450 μl of control solution/50 μl of dH_2_O per vial and diluted 50-fold in isotonic solution. Each well contained 20 μl of the assay with 50 μl isotonic base and 30 μl of either isotonic, dH_2_O to achieve 30% hypotonic solution, dH_2_O with apyrase (1 U/ml, A6535; Sigma–Aldrich) or 10 μM carbenoxolone. Isotonic solution was removed from the cells, and prepared mixes were added to the cells carefully, then ATP was quantified using the Luminoskan Ascent luminometer (Thermo Fisher), integrating over 200 ms and sampling in succession every 10 s for 18 min at 25°C. The ATP release was calculated at different time points indicated and a standard curve transformed the arbitrary light units to ATP concentration, then normalized to mean levels for isotonic solution.

### Data Analysis

Data are reported as mean ± SEM. Analysis was performed in a masked fashion whenever possible. Statistical analysis used a 1-way ANOVA with *post hoc* tests, or paired *t*-test when appropriate. Analysis was performed on ranks when data were not normally distributed. Results with *p* < 0.05 were considered significant.

## Results

### Priming of Inflammasome Genes after Elevation of IOP

**FIGURE 1:**
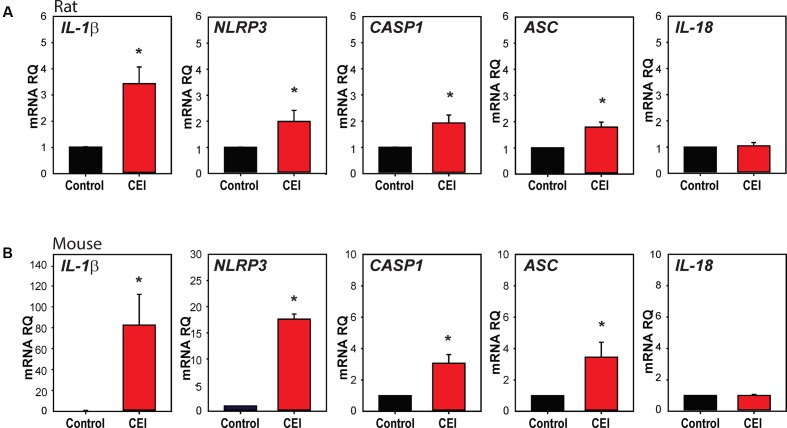
Transient elevation of intraocular pressure primes inflammasome genes in rat and mouse retina. **(A)** Increased expression of inflammasome-associated genes in rat retina after controlled elevation of IOP (CEI). RNA was extracted from the retina soon after IOP returned to baseline following an elevation to 50–60 mmHg for 4 h. The IOP rise led to increased expression of *IL-1*β (^∗^*p* = 0.004, *n* = 10), *NLRP3* (^∗^*p* = 0.045, *n* = 10), *CASP1* (^∗^*p* = 0.014, *n* = 10), and *ASC* (^∗^*p* = 0.008, *n* = 5) as compared to contralateral control eye. There was no detectable rise in *IL-18* (*n* = 5). **(B)** Mouse retina exposed to CEI showed increased expression of *IL-1*β (^∗^*p* = 0.049, *n* = 5), *NLRP3* (^∗^*p* < 0.001, *n* = 3), *CASP1* (^∗^*p* = 0.021, *n* = 3) and ASC (^∗^*p* = 0.029, *n* = 4), but not IL-18 (*n* = 4). Note the scale difference for *IL-1*β. RNA from retina was extracted 22 h after returning IOP to baseline from an elevation to 60 mmHg for 4 h.

We first examined whether mechanical strain primed inflammasome components *in vivo*. IOP was unilaterally elevated in rats to between 50 and 60 mmHg for 4 h using a variant of the Controlled Elevation of IOP (CEI) procedure. RNA was extracted from the retina of treated and contralateral control eyes soon after return of IOP to baseline, and qPCR was used to compare expression of genes associated with the NLRP3 inflammasome. There was a significant elevation in the expression of mRNA for *IL-1*β, *NLRP3*, Interleukin-1 converting enzyme/caspase1 gene (*CASP1*) and Apoptosis-Associated Speck-Like Protein Containing CARD (*ASC*), but not in expression of cytokine *IL-18* (**Figure 1A**).

The procedure was also performed in mice to determine whether the response occurred across species. In material extracted shortly after IOP was returned to baseline, *IL-1*β was elevated moderately (Supplementary Figure S1A). Expression was substantially increased in material extracted 22 h after IOP returned to baseline, with *IL-1*β*, NLRP3*, *CASP1*, and *ASC* levels elevated significantly (**Figure 1B**). The increased expression was greatest for *IL-1*β, with mRNA levels increasing over 80-fold. At neither time point did the CEI procedure elevate message for the pro-apoptotic marker *BAX* (Supplementary Figure S1B), consistent with the lack of cell death found previously with this procedure (Crowston et al., 2015).

Expression was also examined in retinas from Tg-Myoc^Y 437H^ mice; these mice had a sustained, moderate elevation in IOP of 15.5 ± 0.5 mmHg, as compared to 12.2 ± 1.0 in wild type controls at 14–18 months, similar to the IOP difference measured previously at 8 months (Lu et al., 2015). Expression of *IL-1*β mRNA was increased in retinas from Tg-Myoc^Y 437H^ mice compared to littermate controls (Supplementary Figure S1C), but the rise in *NLRP3* or *CASP1* was not significant.

### Inflammasome Priming at Protein Level

**FIGURE 2:**
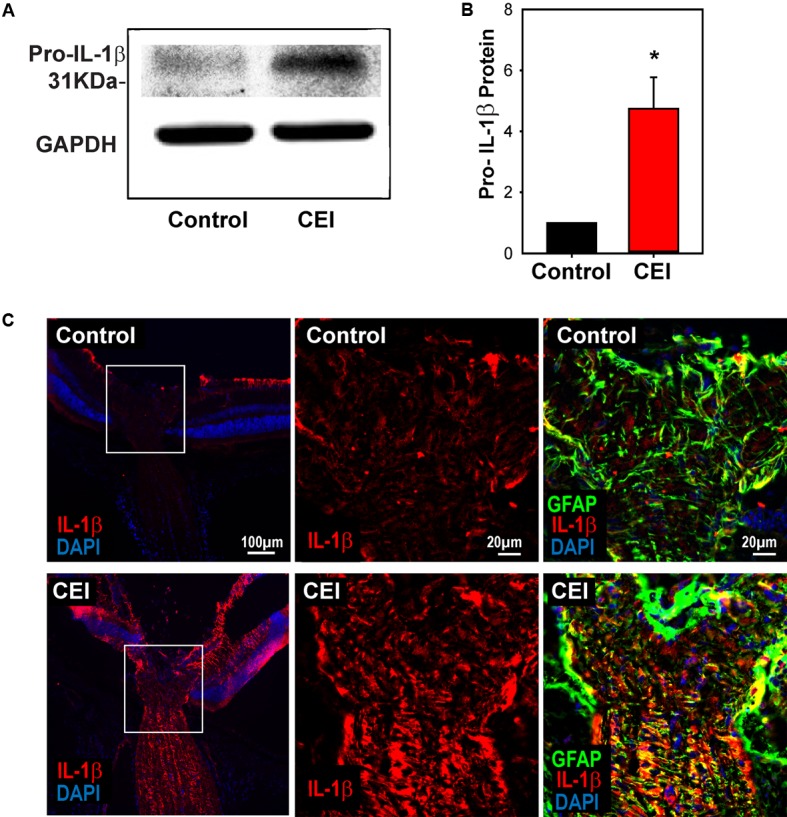
Elevation of IL-1β at the protein level. **(A)** Representative immunoblots from mouse whole retina lysates probed for pro-IL-1β, at the expected 31 kDa size. Protein levels increased in eyes subject to controlled elevation of IOP (CEI) to 60 mmHg for 4 h and sacrificed 22 h after IOP returned to baseline, as compared to the contralateral non-pressurized control eye. Levels of housekeeping protein GAPDH (37 kDa) were similar between conditions. **(B)** Summary of relative protein expression in response to IOP elevation, as quantified with densitometry and normalized to GAPDH levels (^∗^*p* = 0.006, *n* = 5). **(C)** Immunohistochemistry sections of mouse retina stained for IL-1β (red), GFAP (green) and with the nuclear stain DAPI (blue). The top row shows representative images from the non-pressurized eye, while the bottom row is from a contralateral eye exposed to the CEI procedure as in “**(A)**”. Increased staining for IL-1β was apparent in the nerve fiber layer, optic nerve and to a lesser extent throughout the retina (left). Higher magnification of the boxed area shows horizontal bands stained for IL-1β throughout the optic nerve head (center). These bands colocalize with GFAP (right), consistent with optic nerve head astrocytes.

Given that the elevation of *IL-1*β was substantially greater than that of other inflammasome components, further efforts were focused on this cytokine. Immunoblots were performed to probe for pro-IL-1β protein to confirm the mRNA results. Expression of 31 kDa pro-IL-1β protein was significantly elevated in mouse eyes when examined 22 h after IOP elevation (**Figures 2A,B**). Immunohistochemistry was used to localize the rise in IL-1β induced by IOP elevation. Staining for IL-1β was low under control conditions, but increased substantially in eyes exposed to IOP elevation (**Figure 2C** and Supplementary Figure S2). The increased staining was greatest in the nerve fiber bundle of the retina and throughout the optic nerve. Closer inspection of the staining pattern in the optic nerve head showed increased expression of IL-1β in bands through the optic nerve head that colocalized with GFAP, suggesting IL-1β expression was increased in optic nerve head astrocytes.

### P2X7 Receptor Is Involved in IL-1β Priming *In Vivo*

**FIGURE 3:**
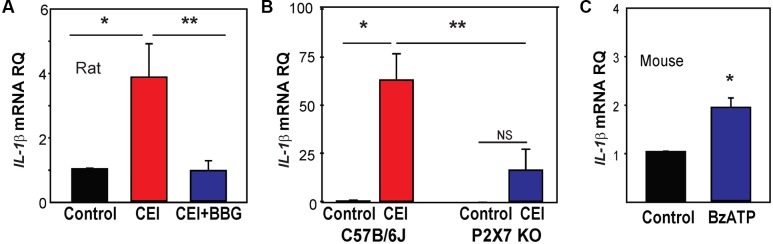
Involvement of the P2X7 receptor in inflammasome priming *in vivo.* **(A)** The pressure-dependent rise in *IL-1*β mRNA in rat retinas exposed to moderate elevation of IOP (CEI) was not present following injection of the P2X7 receptor antagonist BBG. Data are expressed as relative gene expression in the non-pressurized (Control) vs pressurized retina for eyes injected with 0.8% BBG (CEI + BBG) or saline (CEI) 1–3 days before the elevation of IOP to 50 mmHg for 4 h (*n* = 4–5. ^∗^*p* < 0.05 vs. saline pressurized). **(B)** In C57BL/6J mice, the CEI procedure increased retina levels of *IL-1*β mRNA relative to contralateral untreated eyes (^∗^*p* = 0.018). In P2X7 knockout mice, the elevation in IOP did not significantly (NS) increase levels of *IL-1*β. Levels of *IL-1*β mRNA in pressurized eyes of P2X7 knockout mice were significantly less than in wild type pressurized eyes (^∗∗^*p* = 0.036). Data are expressed as gene expression of untreated eyes (Control) relative to pressurized eyes (CEI). Retina including optic nerve head was extracted 22 h after returning IOP to baseline from an elevation to 60 mmHg for 4 h (*n* = 4). **(C)** Intravitreal injection of P2X7 agonist BzATP was sufficient to increase levels *IL-1*β mRNA in mouse retina when extracted 24 h after injection. Data are expressed as relative gene expression of contralateral non-injected eye (Control) vs. injected eye (BzATP; ^∗^*p* < 0.01, *n* = 3).

We hypothesized that the increased expression of following IOP elevation might relate to the release of ATP and autostimulation of P2X7 receptors found in optic nerve head astrocytes exposed to mechanical strain (Beckel et al., 2014). This theory was supported by recent findings implicating the P2X7 receptor in the mechanosensitive upregulation of cytokines IL-3 (Lim et al., 2016) and IL-6 (Lu et al., 2017) in the retina. To determine if the P2X7 receptor was involved in IOP-sensitive priming of IL-1β, the P2X7 receptor antagonist BBG (0.8%) was injected intravitreally 1–3 days before the IOP rise, then retinas were collected 22 h after the IOP returned to baseline. Pretreatment with BBG prevented the upregulation of *IL-1*β mRNA triggered by IOP elevation (**Figure 3A**).

P2X7 receptor involvement was examined further by evaluating *IL-1*β mRNA levels in P2X7^-/-^ mice. Elevation of IOP significantly increased *IL-1*β mRNA levels in control C57BL/6J mouse eyes, but not P2X7^-/-^ mice (**Figure 3B**). A similar reduction was observed in levels of NLRP3 in tissue from the P2X7^-/-^ mice (Supplementary Figure S3A). P2X7 receptor stimulation was itself sufficient to increase *IL-1*β expression; the P2X7 agonist BzATP was injected intravitreally in C57BL/6J mice with sterile saline injected into the contralateral eye as a control. Retinas collected 1 day after the injections showed that BzATP significantly increased the expression of *IL-1*β (**Figure 3C**). The effect of BzATP on other inflammasome genes was much smaller (Supplementary Figure S3B). Together, the data implied the P2X7 receptor was priming *IL-1*β in response to mechanical strain *in vivo*.

### Mechanical Strain Primes Inflammasome Genes in Isolated Astrocytes

The optic nerve head has been identified as a focal center of mechanical strain that accompanies IOP elevation (Burgoyne et al., 2005; Downs et al., 2008), with optic nerve head astrocytes involved in several signaling pathways implicated in glaucomatous pathology (Hernandez, 2000; Morgan, 2000; Tehrani et al., 2016). Mild stretch to optic nerve head astrocytes leads to a release of ATP and autostimulation of P2X7 receptors (Beckel et al., 2014), and the immunohistochemical staining in **Figure 2C** indicated an increase in IL-1β in optic nerve head astrocytes. As such, the mechanosensitive priming of *IL-1*β and the contribution of the P2X7 receptor to this priming was examined further in isolated optic nerve head astrocytes.

**FIGURE 4:**
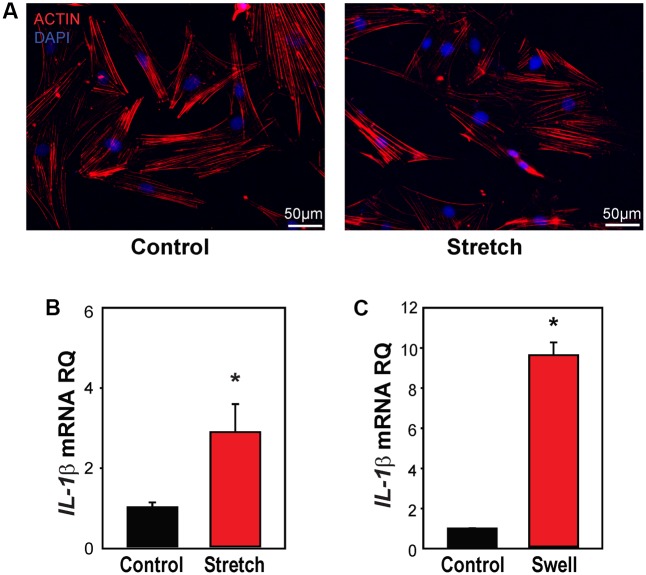
Mechanical strain primes *IL-1*β in optic nerve head astrocytes. **(A)** Astrocytes plated on silicon substrates and fixed after 4 h in control conditions (left, Control) or after 16% cyclical strain at 0.3 Hz (right, Stretch). Staining for actin with phalloidin (red) showed no obvious changes to the cytoskeleton. **(B)** Application of 16% cyclical strain for 4 h increased expression of *IL-1*β mRNA (*n* = 5, ^∗^*p* < 0.03). **(C)** Astrocytes exposed to moderate swelling induced by 30% hypotonicity (Swell) showed increased expression of *IL-1*β mRNA relative to untreated cells maintained in isotonic solution (Control; *n* = 3, ^∗^*p* = 0.009).

Primary rat optic nerve head astrocytes were plated on a silicon sheet and subjected to 16% strain at 0.3 Hz for 4 h. Cells subjected to this stretch protocol looked identical to control cells on a macroscopic level, with very similar patterns of actin staining (**Figure 4A**). The level of *IL-1*β mRNA was significantly increased in stretched cells (**Figure 4B**). Expression of *IL-1*β was also elevated by applying strain to the cells by swelling in a 30% hypotonic solution (**Figure 4C**). The rise in other inflammasome genes induced by swelling astrocytes was variable, with small increases in *NLRP3*, *ASC*, and *IL-18*, but not *CASP1* (Supplementary Figure S4).

### ATP Release through Pannexin Channels Required for Mechanosensitive Priming of IL-1β in Astrocytes

**FIGURE 5:**
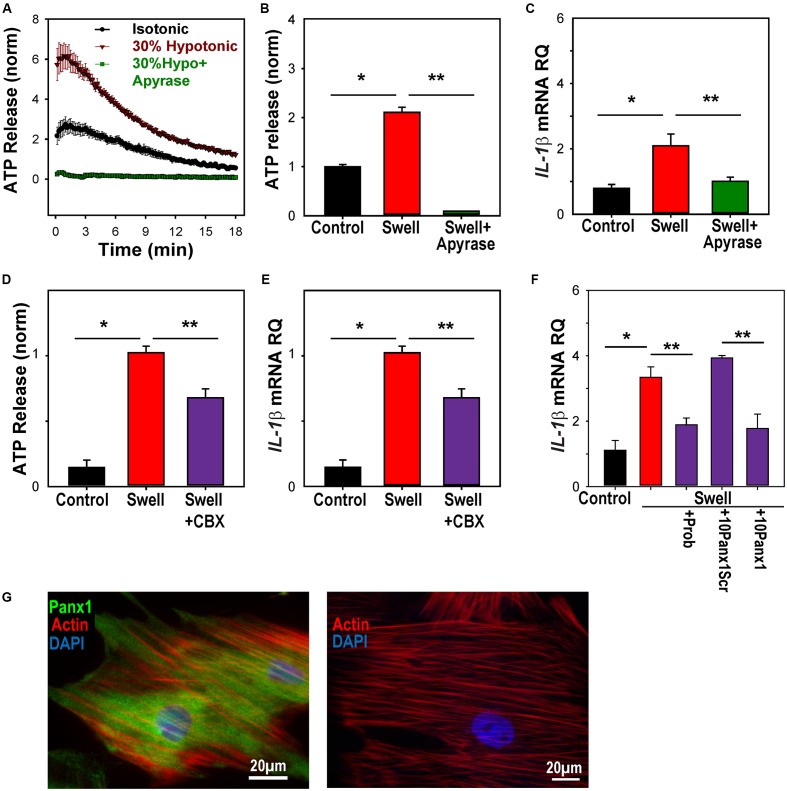
ATP release through pannexin channels required for mechanosensitive priming of *IL-1*β in astrocytes. **(A)** Swelling rat astrocytes in hypotonic solution led to a release of ATP into the extracellular medium, as detected by the luciferin/luciferase assay. The ATP hydrolase apyrase (1 U/ml) substantially reduced the response. Symbols represent mean ± SEM, *n* = 10. **(B)** Quantification of extracellular ATP levels 18 min after exposure to solutions (^∗^Swell vs. Control isotonic, *p* < 0.05, ^∗∗^ Swell vs Swell+Apyrase, *p* < 0.05, *n* = 10). **(C)** Swelling astrocytes in the presence of apyrase also prevented the rise in *IL-1*β mRNA (^∗^*p* = 0.02 Swell vs control, ^∗∗^*p* = 0.03 Swell vs Swell+Apyrase, *n* = 3). **(D)** The swelling-induced release of ATP was inhibited by pannexin channel blocker carbenoxolone (CBX, 10 μM, ^∗^*p* < 0.05, Swell vs. control, ^∗∗^*p* < 0.05 Swell vs. Swell+CBX, *n* = 20, normalized to swell). **(E)** The swelling-induced rise in *IL-1*β mRNA was also inhibited by 10 μM carbenoxolone (^∗^*p* < 0.05, Swell vs. control, ^∗∗^*p* < 0.05 Swell vs Swell+CBX, *n* = 7, normalized to swell). **(F)** Pannexin blocker probenecid (Prob, 1 mM) reduced the swelling-induced rise of *IL-1*β in astrocytes (*p* = 0.029). The specific peptide blocker ^10^Panx1 (100 μM) reduced the expression of *IL-1*β as compared to the scrambled peptide control (10panxscr, ^∗^*p* = 0.003). Swelling alone raised *IL-1*β (*p* = 0.03, *n* = 3 for all). **(G)** Left: Astrocytes stained for panx1 (green), actin (red) and DAPI (blue). Right: No signal was detected in the absence of panx1 antibody.

The measurements of *IL-1*β mRNA from optic nerve head astrocytes *in vitro* allowed further investigation of the mechanisms linking mechanical strain to *IL-1*β upregulation. First, the ability of astrocytes to release ATP when swollen, and of the soluble ectoATPase apyrase to prevent the extracellular elevation in ATP was confirmed (**Figures 5A,B**). The ability of apyrase to prevent the swelling-induced rise in *IL-1*β expression supported a role for extracellular ATP in this pathway (**Figure 5C**).

Previous work suggests that pannexin hemichannels are a conduit for the mechanosensitive release of ATP from these cells (Beckel et al., 2014). Carbenoxolone is reported to be relatively specific for pannexin channels at 10 μM (Bruzzone et al., 2005), and this concentration led to a moderate, but significant reduction in the swelling-induced release of ATP (**Figure 5D**). This concentration of carbenoxolone reduced the rise in *IL-1*β mRNA expression by a similar amount (**Figure 5E**). The swelling-induced rise in *IL-1*β was also blocked by blocked by probenecid and the peptide blocker ^10^panx1, while the scrambled peptide control had no effect on expression (**Figure 5F**). The expression of pannexin 1 in these astrocytes (**Figure 5G**), combined with the reduction by three pannexin blockers, implicated pannexins in the *IL-1*β response.

### P2X7 Receptor Necessary and Sufficient for Mechanosensitive Priming of IL-1β *In Vitro*

**FIGURE 6:**
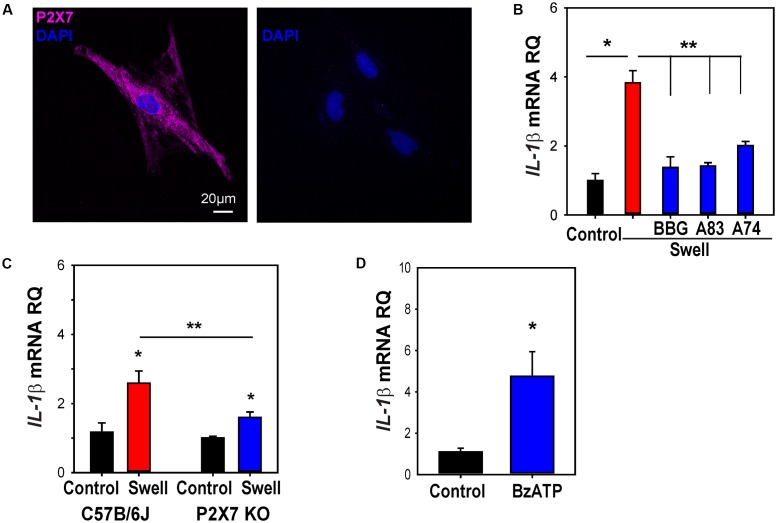
P2X7 receptor involved in priming of *IL-1*β in astrocytes. **(A)** Immunocytochemistry showing expression of the P2X7R in cultured optic nerve head astrocytes (left). No signal was detected in the absence of the primary antibody (right). **(B)** The swelling-induced rise in *IL-1*β mRNA was inhibited by P2X7R antagonists BBG (10 μM), A839977 (50 nM) and A740003 (5 μM). Cells were pretreated with drugs for 1 h before swelling (^∗^*p* < 0.001 Swell vs. control, ^∗∗^*p* < 0.001 Swell vs. Swell+drugs, *n* = 4). **(C)** The swelling-induced rise in *IL-1*β was reduced in astrocytes from P2X7^-/-^ mice as compared to C57BL/6J mice. Data are expressed relative to the matched control group (^∗^*p* < 0.01, ^∗∗^*p* = 0.026, *n* = 6). **(D)** Application of BzATP (400 μM) for 4 h increased *IL-1*β expression (^∗^*p* < 0.01, *n* = 7).

ATP released after swelling can autostimulate astrocytes, with P2X7 antagonists blocking the rise in cytoplasmic Ca^2+^ induced by swelling (Beckel et al., 2014). To determine whether this autostimulation contributed to the priming of IL-1β, the effect of P2X7 antagonists on the swelling-dependent rise in IL-1β was examined. The expression of the P2X7 receptor in astrocytes was confirmed using immunocytochemistry (**Figure 6A**). Three P2X7 antagonists, BBG, A839977, and A740003, significantly prevented the mechanosensitive *IL-1*β priming in optic nerve astrocytes (**Figure 6B**). To support this pharmacological identification of the P2X7 receptor, experiments were pursued on astrocytes isolated from C57Bl/6J mice and P2X7^-/-^ mice. PCR confirmed the absence of the P2X7 receptor message in astrocytes obtained from knockout mice while immunocytochemistry supported the absence of P2X7 protein (Supplementary Figures S5A,B). The increased in expression of *IL-1*β mRNA after 4 h of swelling was significantly lower in astrocytes from the P2X7^-/-^ mice as compared to the C57BL/6J mice (**Figure 6C**). Treatment of astrocytes with the P2X7 receptor agonist BzATP was sufficient to upregulate *IL-1*β mRNA (**Figure 6D**). Similar results were found with *NLRP3*; the response was reduced in astrocytes from P2X7^-/-^ mice (Supplementary Figure S5C), while addition of BzATP induced a significant, albeit small, rise in *NLRP3* (Supplementary Figure S5D).

### NFκB Is Involved in Priming of NLRP3 and IL-1β after Mechanical Strain

**FIGURE 7:**
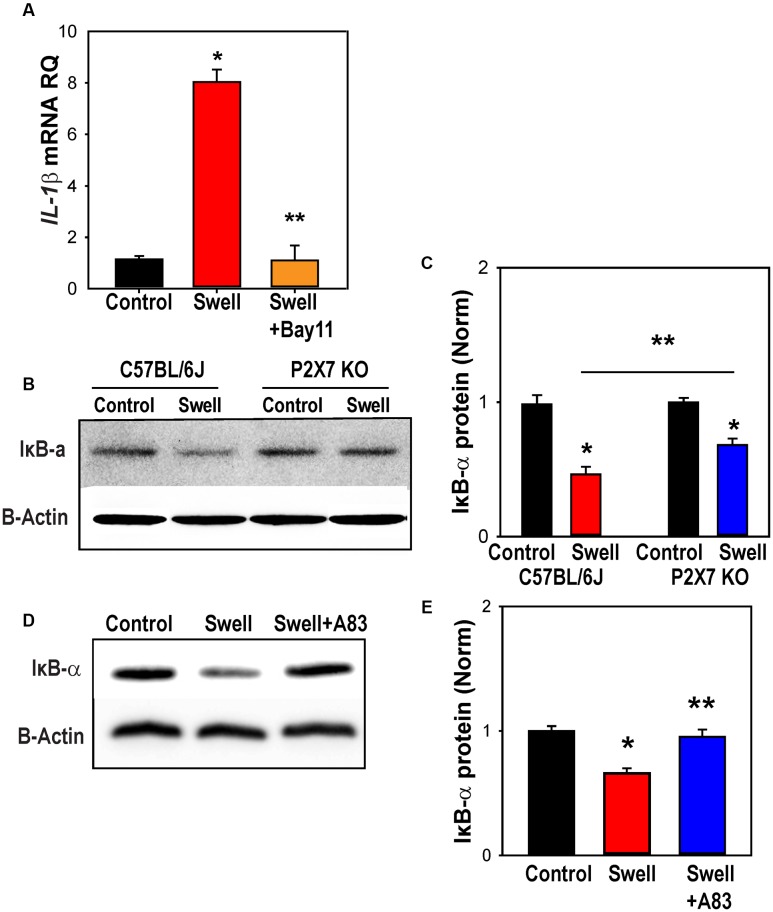
NFκB is involved in inflammasome priming after mechanical strain. **(A)** NFκB inhibitor Bay11-7082 (Bay11, 4 μM) prevented *IL-1*β upregulation in rat astrocytes. Bay11-7082 was present for 1 h before and during the 4 h swelling (^∗^*p* < 0.001 Control vs. Swell, ^∗∗^*p* < 0.001, Swell vs. Swell+Bay11; *n* = 4). **(B)** Representative immunoblots from mouse optic nerve head astrocyte lysates from control C57BL/6J and P2X7^-/-^ mice probed for IκB-α (39 kDa) and housekeeping protein β-actin (42 kDa). Expression of IκB-α was reduced following 4 h of swelling in control astrocytes, consistent with the activation of NFκb. **(C)** Summary of relative IκB-α protein expression from experiments illustrated in panel B quantified with densitometry. The effect of swelling on IκB-α was significantly less in astrocytes from P2X7^-/-^ mice (^∗^*p* < 0.001 Swell vs. Control C57BL/6J, ^∗^*p* = 0.011 Swell vs. Control P2X7^-/-^, ^∗∗^*p* = 0.038 Swell C57BL/6J vs. Swell P2X7^-/-^; *n* = 3). **(D)** Representative immunoblots from mouse optic nerve head astrocyte lysates from control mice probed for IκB-α (39 kDa) and housekeeping protein β-actin (42 kDa). The reduction in IκB-α triggered by swelling was reduced in the presence of P2X7R antagonist A839977 (100 nM). **(E)** Mean densitometry values for IκB-α protein expression from immunoblots like those in “**(D)**” (^∗^*p* = 0.002, ^∗∗^*p* = 0.004; *n* = 4).

While many different transcription factors could be involved in the upregulation of IL-1β, we focused on the contribution of NFκB, as it is linked to the transcription of inflammasome genes including IL-1β (Cogswell et al., 1994) and can be activated by P2X7 stimulation (Liu et al., 2011). The NFκB inhibitor Bay 11-7082 prevented the swelling-induced upregulation of *IL-1*β in rat astrocytes (**Figure 7A**). Upregulation of *NLRP3* was similarly blocked by Bay 11-7082 (Supplementary Figure S6).

To confirm a role for NFκB in the transcriptional changes, levels of nuclear factor of kappa light polypeptide gene enhancer in B-cells inhibitor, alpha (IκBα) in extracts from control and swollen astrocytes from C57BL/6J were probed with immunoblots. The reduction in Iκbα levels corresponds to activation of NFκB (Finco and Baldwin, 1995). Swelling reduced levels Iκbα in astrocytes from control mice, but not in cells from P2X7^-/-^ mice (**Figure 7B**). Quantification showed the reduction in Iκbα induced by swelling was significantly less in astrocytes from P2X7^-/-^ mice (**Figure 7C**). The P2X7 receptor antagonist A839977 also reduced the ability of swelling to activate IκBα (**Figures 7D,E**), supporting a role for the P2X7 receptor in the swelling-dependent activation of NFκB.

## Discussion

This study suggests that mechanical strain can increase expression of certain components of the NLRP3 inflammasome in neural tissue and identifies a role for ATP release and the P2X7 receptor in this priming. The cytokine IL-1β was linked through this pathway most strongly, with supportive evidence for upregulation of NLRP3. Given that priming is the initial step in NLRP3 inflammasome involvement, this study implicates a role for the P2X7 receptor in linking mechanical strain to innate immune responses in neural tissues.

### Role of Purinergic Signaling

Evidence linking the P2X7 receptor with priming of IL-1β comes from *in vivo* and *in vitro* assays of mRNA and protein. The P2X7 antagonist BBG prevented the rise in *IL-1*β expression *in vivo* in rat retinas exposed to a transient rise in IOP. The rise in IL-1β following transient IOP increase was significantly less in P2X7^-/-^ mice as compared to control, while the rise in *IL-1*β expression following intravitreal injection of P2X7 agonist BzATP suggests receptor stimulation is sufficient to increase *IL-1*β expression.

*In vitro* work using isolated astrocytes provides additional support and implicates the P2X7 receptor more specifically, with the use of more selective antagonists A839977 and A740003 (Honore et al., 2006, 2009), in addition to BBG. The pressure-induced rise in IL-1β expression was prevented by these agents and was not present in P2X7^-/-^ mice, while the P2X7 agonist BzATP was sufficient to elevate IL-1β. Together, the combined evidence from pharmacological and genetic methods, and in both isolated astrocytes and whole retina, strongly implicates a role for the P2X7 receptor in priming of IL-1β. The identification of a P2X7 receptor contribution in both rats and mice suggests receptor involvement may be widespread, particularly given the differences in the receptor across these species (Donnelly-Roberts et al., 2009).

**FIGURE 8:**
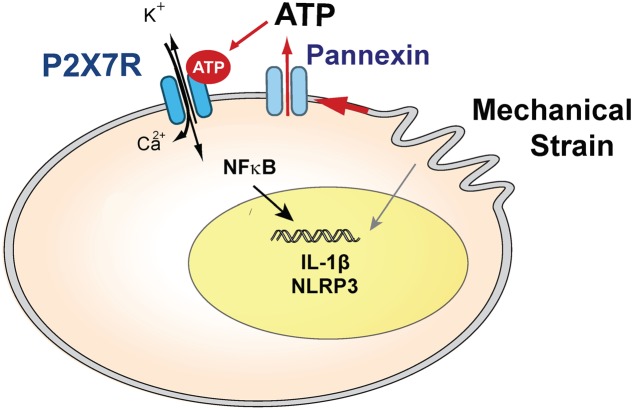
Model summarizing the hypothesized role of the P2X7 receptor in the priming of inflammasome genes after mechanical strain, which leads to ATP release through pannexin hemichannels. Extracellular ATP autostimulates P2X7 receptors, leading to NFκB activation and transcriptional elevation of *IL-1*β and *NLRP3* in optic nerve head astrocytes. Swelling may also activate inflammasome genes through other pathways.

Involvement of extracellular ATP in IL-1β priming was supported by the ability of the soluble ectoATPase apyrase to block gene upregulation. The ability of pannexin channel blockers carbenoxolone, probenecid and the ^10^panx1 peptide to prevent a rise in *IL-1*β strongly implicates the release of ATP through the hemichannel in priming, as these drugs inhibited the ATP release induced by astrocyte swelling (Beckel et al., 2014). The partial block by carbenoxolone is consistent, as this concentration shows specificity for pannexins over connexins while not providing a complete block, although it may also reflect a contribution from an alternative efflux mechanism. Overall, these studies suggest a model in which mechanical strain leads to release of ATP through pannexin hemichannels, autostimulation of the P2X7 receptor and subsequent priming of IL-1β (**Figure 8**).

### Transcription Factors and Gene Variation

The transcription factor NFκB was implicated in the upregulation of IL-1β and NLRP3 in astrocytes. Increased expression of both genes in swollen astrocytes was blocked by NFκB antagonist Bay 11-7082. The P2X7 receptor was implicated by data showing the swelling-dependent decreased in NFκB inhibitor Iκbα was reduced in astrocytes from P2X7^-/-^ mice and by the P2X7 receptor antagonist A839977. Elevation of hydrostatic pressure leads to translocation of NFκB to the nucleus in retinal astrocytes (Sappington and Calkins, 2006), while NFκB regulates transcription of NLRP3 and IL-1β in other cells (Cogswell et al., 1994; Lawrence, 2009; Boaru et al., 2015). The P2X7 receptor has been shown to activate NFκB through contact with MyD88 in HEK cells (Liu et al., 2011). This makes the activation of NFκB by P2X7 a likely route to connect mechanical strain with increased expression of IL-1β and NLRP3. While the residual activation in astrocytes from P2X7^-/-^ mice may reflect the involvement of other pathways, the presence of P2X7 splice variants may provide additional possibilities (Masin et al., 2012).

The increase in *IL-1*β in response to mechanical strain was particularly consistent, observed both in rat and mouse *in vivo* models, and in cultured astrocytes from rat and mouse tissues; the increase in the 31 kDa pro-form in immunoblots confirmed this on a protein level. While expression of *NLRP3, CASP1*, ASC, and *IL-18* were all increased by some model of mechanical strain, the effects in these genes were less consistent. Some of this variation may have been time-dependent, as the expression of most genes was substantially larger in mouse retina 22 h after IOP elevation was returned to baseline. The diverse responses to swelling and stretching were not unexpected given that *IL-1*β*, IL-18, NLRP3, CASP1*, and *ASC* are all regulated by a different combination of transcription factors. Regardless, the priming of *IL-1*β and *NLRP3* may be rate limiting in inflammasome activation, as CASP1 and IL-18 are constitutively expressed in monocytes and epithelial cells (Thornberry et al., 1992; Dinarello, 2007).

### Contribution of Astrocytes

The P2X7 receptor was implicated in priming *IL-1*β in both *in vivo* experiments, where material from the entire retina was analyzed, and during the *in vitro* experiments using isolated optic nerve head astrocytes. These optic nerve head astrocytes make up a small proportion of retinal material, however, and increased staining for IL-1β in various parts of the retina after elevated IOP suggests additional cell types may contribute to the retinal response. For example, our staining was consistent with increased expression in Muller glial cells. Neuronal involvement is also likely; the increased staining mentioned above is supported by recent results showing increased *IL-1*β expression in isolated retinal ganglion cells exposed to stretch (Lim et al., 2016); these neurons release ATP and autostimulate their P2X7 receptors, suggesting a parallel pathway may be involved (Xia et al., 2012). However, the optic nerve head is a focal center of mechanical strain in the glaucomatous eye (Burgoyne et al., 2005), and astrocytes from patients showed morphological changes before marked loss of retinal ganglion cells (Lye-Barthel et al., 2013). The astrocytes express mechanosensitive channels (Choi et al., 2015) and contribute to the inflammatory response in glaucomatous eyes (Johnson and Morrison, 2009). As such, the identification of the P2X7 receptor linking mechanical strain to inflammasome priming in optic nerve head astrocytes is particularly relevant.

### ATP as Endogenous Trigger Linking Mechanical Strain to Inflammation in Neural Tissues

Inflammation has emerged as a critical component of chronic neurodegeneration, with the NLRP3 inflammasome a major contributor (Freeman and Ting, 2016). While priming of the NLRP3 inflammasome traditionally has been attributed to stimulation of toll-like receptors (Patel et al., 2017), these receptors are primarily activated by pathogens, and the endogenous triggers linking neural insult to inflammasome priming are largely unknown.

Our identification of the P2X7 receptor as a trigger for NLRP3 inflammasome priming in the retina builds on evidence linking mechanical strain to aberrant purinergic signaling in the retina and allows this endogenous trigger to be placed in a physiological context. Extracellular ATP is elevated after increased IOP in bovine mouse, rat, primate and human samples (Zhang et al., 2007; Reigada et al., 2008; Lu et al., 2015). Stimulation of P2X7 receptors can damage retinal ganglion cells *in vitro* and *in vivo* (Zhang et al., 2005; Hu et al., 2010), and ATP release through pannexin hemichannels following mechanical strain can autostimulate P2X7 receptors on optic nerve head astrocytes (Beckel et al., 2014). As pannexin hemichannels are upregulated by prolonged stretch *in vitro* and *in vivo*, this provides a source of the sustained extracellular ATP found in the chronic glaucoma models. The present study suggests this may also provide a mechanism for chronic priming of inflammasome genes.

The P2X7 receptor is traditionally known for its ability to activate the NLRP3 inflammasome following the efflux of K^+^ through the open channel (Katsnelson et al., 2015). The present study identifies a novel role for the P2X7 receptor in the priming of IL-1β and NLRP3. The ability of one receptor to mediate both steps of inflammasome involvement identifies a potentially central role for purinergic signaling in the link between mechanical strain and innate inflammation in neural tissues. Future studies focused on the contributions of the P2X7 receptor to inflammasome activation following its role in priming will clarify how this “double punch” impacts the inflammatory state of the retina.

The P2X7 receptor is implicated in multiple forms of neural degeneration (Sanderson et al., 2014; Sperlagh and Illes, 2014). Neuronal loss after axotomy was reduced in P2X7 knock out mice (Nadal-Nicolas et al., 2016) while the receptor is also implicated in Alzheimer’s disease (Miras-Portugal et al., 2015). Activation of the P2X7 receptor was implicated in a study of traumatic brain injury, with expression of IL-1β reduced in knock out mice (Kimbler et al., 2012). The present study identifies a role of the receptor in priming of IL-1β and other genes involved with the NLRP3 inflammasome, suggesting a central role in the inflammasome activation.

Portions of this work have previously been presented in abstract form (Lu et al., 2013; Albalawi et al., 2016; Mitchell et al., 2016, 2017).

## Availability of Data and Materials

All readily reproducible materials described in the manuscript, including new software, databases and all relevant raw data will be freely available to any scientist wishing to use them.

## Author Contributions

FA helped design the study, carried out experiments on elevation of IOP in mice and rats and experiments on isolated astrocytes. WL helped design the study and developed the protocols for *in vivo* experiments and carried out some rat IOP experiments, JB and JL performed *in vitro* experiments on rat optic nerve head astrocytes. SM helped in the writing of the manuscript. CM conceived of the study, and participated in its design and coordination and helped to draft the manuscript. All authors read, edited and approved of the final manuscript.

## Conflict of Interest Statement

The authors declare that the research was conducted in the absence of any commercial or financial relationships that could be construed as a potential conflict of interest.
