# Supplementary material for: The P2X7 Receptor Primes IL-1β and the NLRP3 Inflammasome in Astrocytes Exposed to Mechanical Strain
Source: Front Cell Neurosci. 2017 Aug 8;11:227. doi: 10.3389/fncel.2017.00227 (PMC5550720; doi:10.3389/fncel.2017.00227)
Supplement: Supplementary file 1 [file Presentation_1.PDF]

Supplementary Material

Title: The P2X7 receptor primes IL-1β and the NLRP3 inflammasome in astrocytes exposed to mechanical strain

Authors: Farraj Albalawi, Wennan Lu, Jonathan M. Beckel, Jason C. Lim, Stuart A. McCaughey, Claire H. Mitchell*

*** Correspondence:** Corresponding Author: chm@upenn.edu

Figure S1

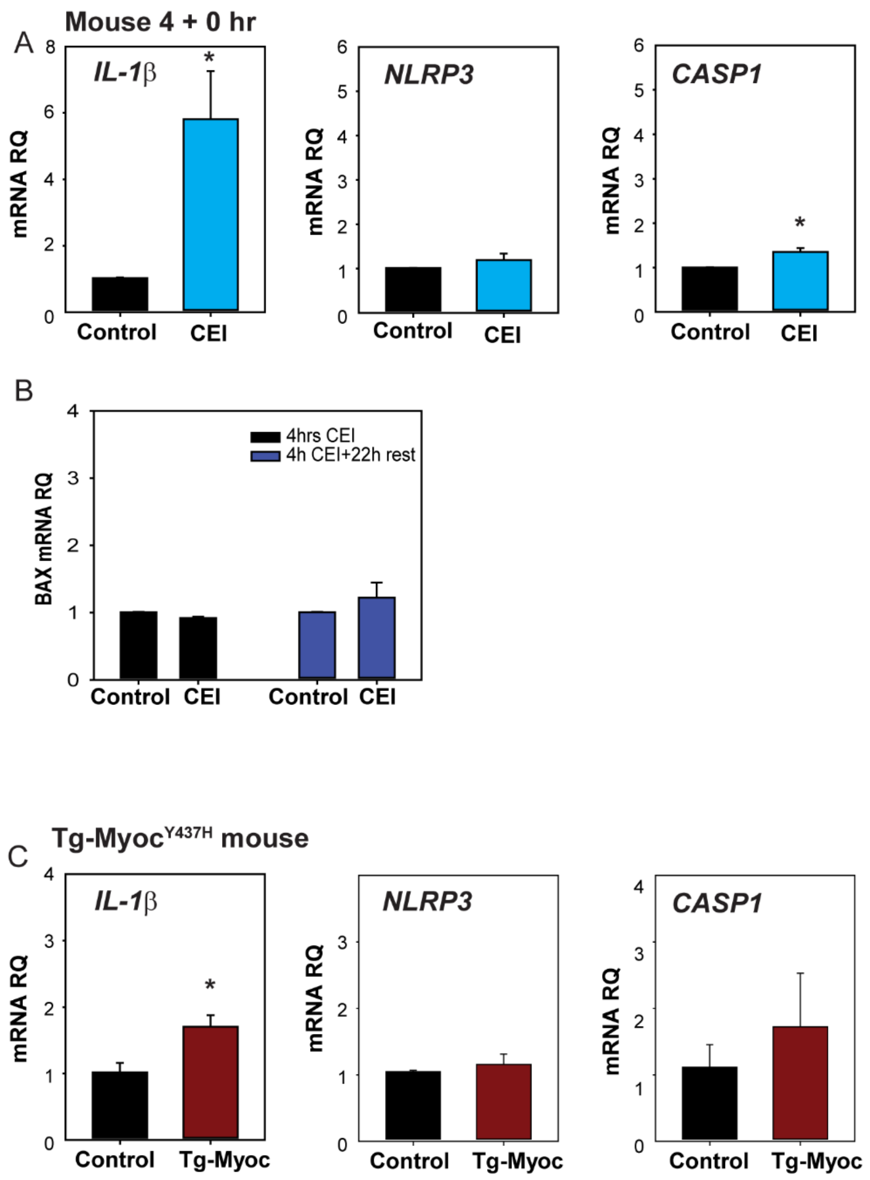

Figure S1

A. Expression of genes obtained from mouse retinas (including the optic nerve head material) immediately after a 4 hr elevation of IOP to 60 mmHg as compared to levels in the contralateral unpressurized eye. Expression of *IL-1β* (*p=0.006, n=6) and *CASP1* rose (*p = 0.002, n=6) while expression of *NLRP3* did not change.

B. The CEI procedure did not increase the apoptosis regulator *BAX* at the mRNA level immediately after the transient elevation of IOP to 60 mmHg for 4 hrs or if allowed to rest for 22hrs before extraction (n=3).

C. In retina obtained from 14-18 month old Tg-Myoc^{Y437H} mice (Tg-Myoc), *IL-1β* mRNA expression was greater than in controls (*p=0.02, n=3). Neither the rise in *NLRP3* nor *CASP1* were significant (n=3).

Figure S2

A. Negative controls of IL-1b and GFAP staining

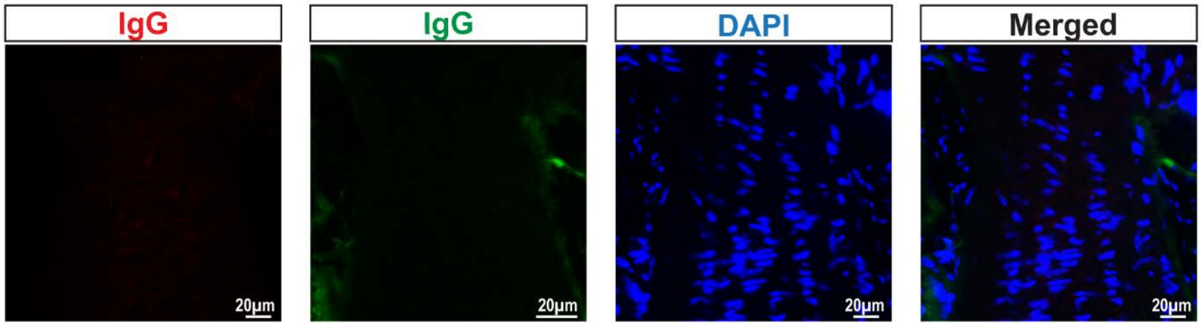

Figure S2

Representative negative controls for the immunohistochemistry of mouse retina treated with the goat IgG then the secondary donkey anti-goat Alexa555-conjugated and donkey anti-mouse IgG Alexa-Fluor 488 in parallel to the immunostaining in Fig 2. ImageJ was used to modify intensity and combine pseudocolored images, with parallel processing for all images in Figs. 2 and S2. Scale bar = 20μm.

Figure S3

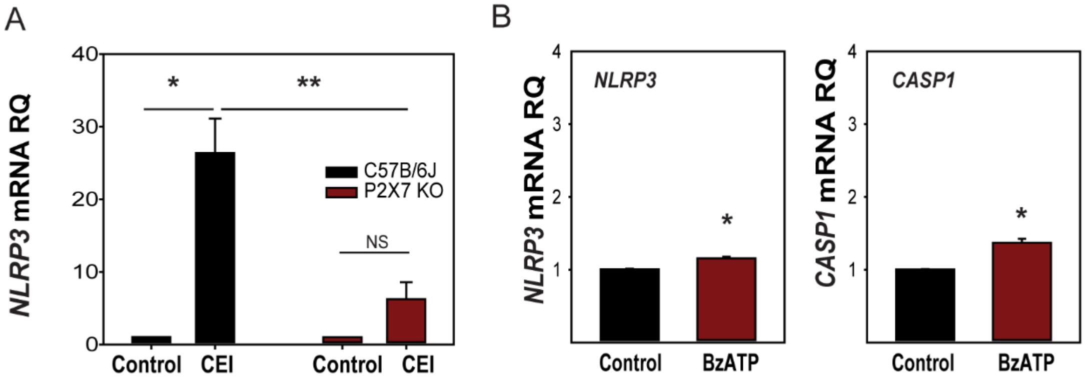

Figure S3

A. In C57BL/6J mice, transient elevation of IOP to 60 mmHg for 4 hrs raised retina levels of NLRP3 mRNA relative to paired untreated eyes (*p<0.01). In P2X7 knockout mice, the elevation in IOP did not significantly increase levels of NLRP3. Levels of NLRP3 mRNA from P2X7 knockout mice pressurized eyes were significantly less than in wildtype pressurized eyes (**p<0.01). Data are expressed as gene expression of pressurized eyes (CEI) relative to untreated eyes (Control). Retina including optic nerve head was extracted 22 hrs after returning IOP to baseline from an elevation to 60 mmHg for 4 hrs. n=4 in all cases.
B. Intravitreal injection of P2X7R agonist BzATP was sufficient to increase levels of mouse retina mRNA for *NLRP3* and *CASP1*. Data are expressed as relative gene expression of injected eye (BzATP) vs contralateral non-injected eye (Control). n=3 in all cases. *p≤0.01

Figure S4

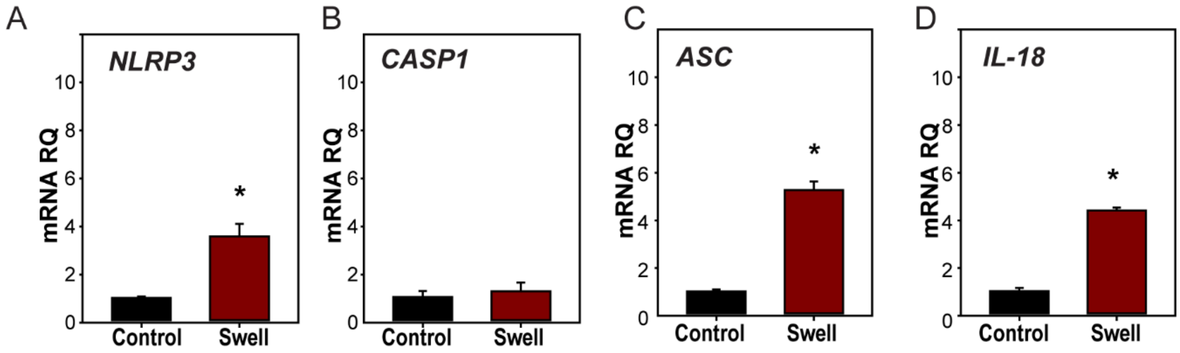

Figure S4

Astrocytes exposed to moderate swelling induced by 30% hypotonicity (Swell) showed increased expression, relative to untreated cells (Control), of mRNA for*NLRP3* (A), *ASC* (C) and *(D) IL-18*, but swelling had no effect on *CASP1* expression (B); n=3 and *p≤0.009 in all cases.

Figure S5

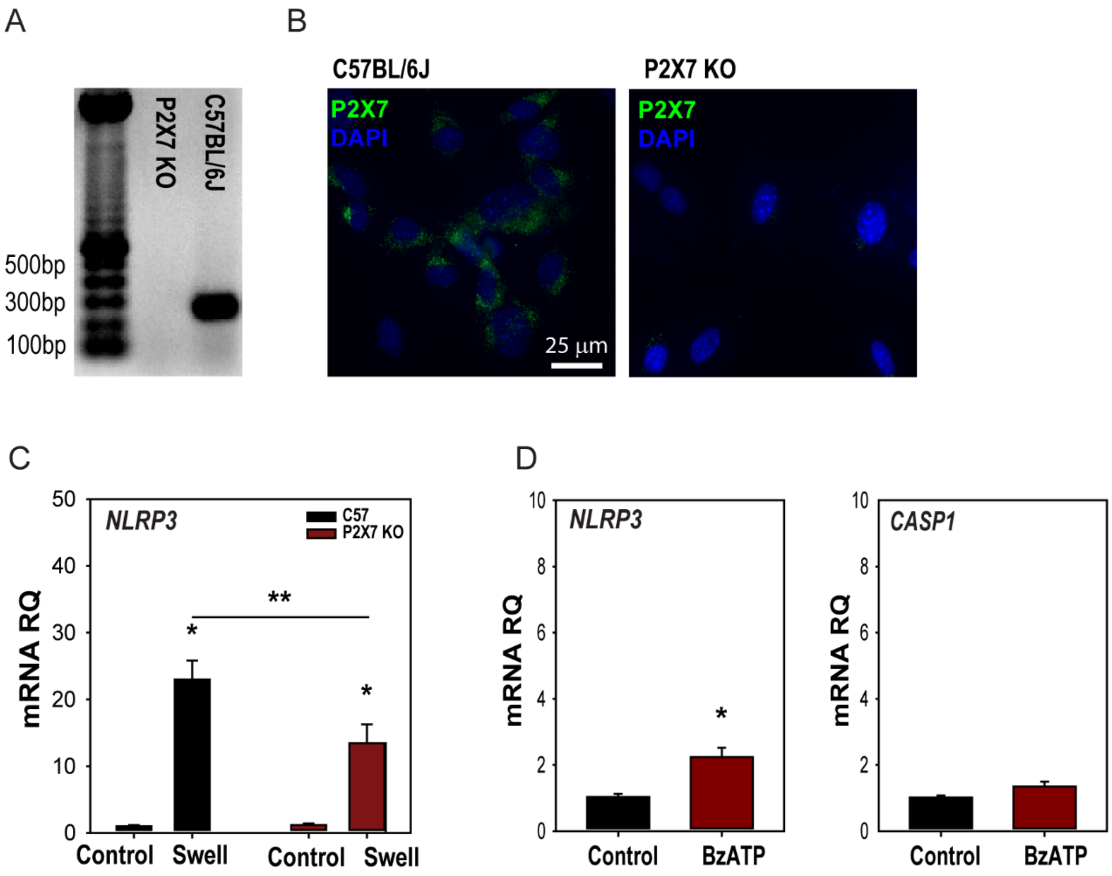

Figure S5

A. PCR of astrocytes obtained from P2X7 knockout and C57BL/6J mice confirmed the absence of the expected 246 bp band in cells from the knockout animals.
B. Immunohistochemistry showing staining for P2X7R in astrocytes cultured from control C57BL6J mice but not from P2X7$^{-/-}$ mice.
C. The swelling-dependent rise in *NLRP3* expression was significantly reduced in astrocytes from P2X7 $^{-/-}$ mice (n=6, *p=0.04, ** p<0.05).
D. The P2X7R agonist BzATP (400 µM) led to a slight increase in *NLRP3* but not *CASP1* (n=7, *p<0.01).

Figure S6

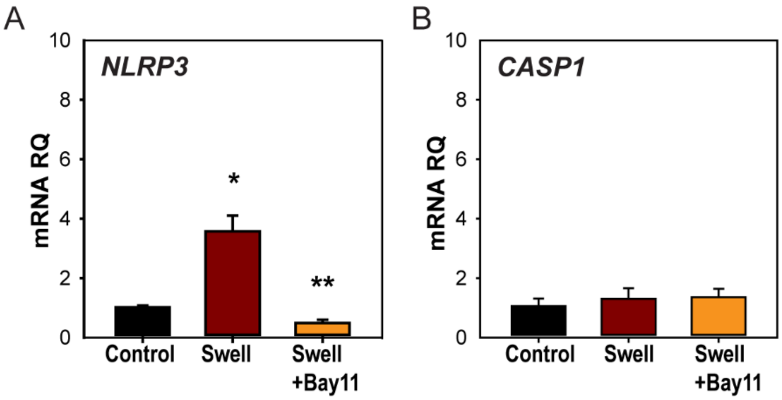

Figure S6

A. The NFkB inhibitor Bay 11-7082 (Bay11, 4μM) reduced *NLRP3* mRNA upregulation in swollen rat astrocytes. Bay11-7082 was present for 1 hr before and during the 4 hr swelling. n=4, *p<0.001 Control vs Swell, **p≤0.001 Swell vs Swell+Bay11.
B. Neither swelling nor Bay 11-7082 had any effect on expression of *CASP1*.
